# Correlation between regional post systolic index and culprit artery in NSTEMI with preserved left ventricular function

**DOI:** 10.1016/j.ahjo.2026.100836

**Published:** 2026-07-08

**Authors:** Aliasghar Farsavian, Maryam Nabati, Mehrdokht Sadrolodabai, Saeed Kavousi

**Affiliations:** a Department of Cardiology, Faculty of Medicine, Cardiovascular Research Center, Mazandaran University of Medical Sciences, Sari, Iran; b Faculty of Medicine, Cardiovascular Research Center, Mazandaran University of Medical Sciences, Sari, Iran

**Keywords:** Coronary artery disease, Deformation, Post-systolic shortening index, Speckle-tracking echocardiography

## Abstract

**Background:**

Patients with non-ST-segment elevation myocardial infarction (NSTEMI) typically present critical arterial stenosis, defined as the stenosis≥70% of the culprit artery. In addition, myocardial shortening that occurs after end-systole, called post-systolic shortening index (PSI), has been acknowledged as a sign of myocardial ischemia, which is easily measurable by the two-dimensional speckle-tracking echocardiography (2D-STE).

**Aim:**

This study was to investigate the correlation between regional average PSI and coronary angiography (CAG)-identified culprit artery among patients with NSTEMI and preserved left ventricular ejection fraction (LVEF).

**Methods:**

Applying a descriptive-correlational research design, the present study was conducted on 54 patients with NSTEMI and LVEF≥50%, admitted to the emergency department of our hospital between 2022 and 2023. All patients underwent a transthoracic 2D-STE within the first day of admission. An 16-left ventricular (LV) segment model of myocardial perfusion territories was then utilized, and regional strain was further explained according to the perfusion territory of each major epicardial artery. Besides, CAG was performed for all patients during 24–48 h of admission. The patients with an identifiable culprit artery on CAG were subsequently divided into two groups based on the location of the culprit artery: Group I (*n* = 28) with the culprit artery within the left anterior descending (LAD) territory, and Group II (*n* = 26) with the culprit artery in the right coronary (RCA) or left circumflex (LCX) artery.

**Results:**

The PSI_LAD_ was found to be higher in Group I than Group II (3.00 [0.33–9.92] vs. 0.63 [0.06–5.50], respectively, *p* = 0.019). The logistic regression analysis (LRA) of different variables additionally revealed that only the average PSI_LAD_ (odds ratio [OR] 1.115, 95% confidence interval [CI]: 1.005–1.239, *p* = 0.04) was independently correlated with the CAG-identified culprit artery in the LAD. Moreover, the receiver operating characteristic (ROC) curve analysis demonstrated that the area under the curve (AUC) for the average PSI_LAD_ to predict the CAG-identified LAD culprit artery was 0.679 (*p* = 0.016; 95% CI [0.540–0.818]).

**Conclusion:**

Regional average PSILAD was independently associated with LAD culprit lesions identified by coronary angiography in a selected population of NSTEMI patients with preserved LVEF. Given the modest diagnostic performance and limited sample size, these findings should be considered exploratory and require validation in larger and more representative NSTEMI cohorts.

## Highlights

• Post-systolic shortening reflects regional myocardial ischemia in NSTEMI.
• Average PSI in LAD territories is higher in LAD culprit lesions.
• Average PSILAD independently predicts angiographic LAD culprit artery.
• PSILAD shows moderate diagnostic accuracy for LAD culprit identification.
• PSI may aid noninvasive localization of culprit vessels in NSTEMI.

## 1 Introduction

With regard to its ever-increasing prevalence and mortality rates in recent years, non-ST-segment elevation myocardial infarction (NSTEMI) accounts for 60–70% of myocardial infarction (MI) events [1]. Patients with NSTEMI typically present critical arterial stenosis, defined as the stenosis≥70% of the culprit artery [2]. In this context, a culprit artery refers to the coronary stenosis recognized to blame for NSTEMI, whose early diagnosis might lead to appropriate treatments [3]. Almost one-third of patients with non-ST-segment elevation acute coronary syndrome (NSTE-ACS) do not require invasive procedures, such as coronary angiography (CAG) and revascularization. However, such patients with normal wall motion and left ventricular ejection fraction (LVEF) pose the biggest challenges in terms of the non-invasive identification of coronary artery lesions. Therefore, much effort has been so far made to find reliable markers to meet early diagnosis and risk stratification in patients with NSTE-ACS [4]. In this line, the speckle-tracking echocardiography (STE) is able to accurately detect some subtle changes in myocardial deformation in patients without visible regional wall motion abnormality (RWMA) [5]. As well, myocardial shortening that occurs after end-systole, called post-systolic shortening index (PSI), can be easily measured by the two-dimensional (2D) STE (2D-STE) modality. This index is a sign of myocardial ischemia as well as a marker of ischemic memory due to its persistence after recovery. Furthermore, this phenomenon may be attributed to the short duration of myocardial stunning [6]. Indeed, post-systolic shortening (PSS) has been suggested as a reliable marker of regional myocardial ischemia and viability. Nevertheless, it is not pathognomonic for myocardial ischemia and may occur in healthy individuals [7]. The aim of this study was to investigate the correlation between regional average PSI and CAG-identified culprit artery among patients with NSTEMI and preserved LVEF.

## 2 Methods

**Fig. 1:**
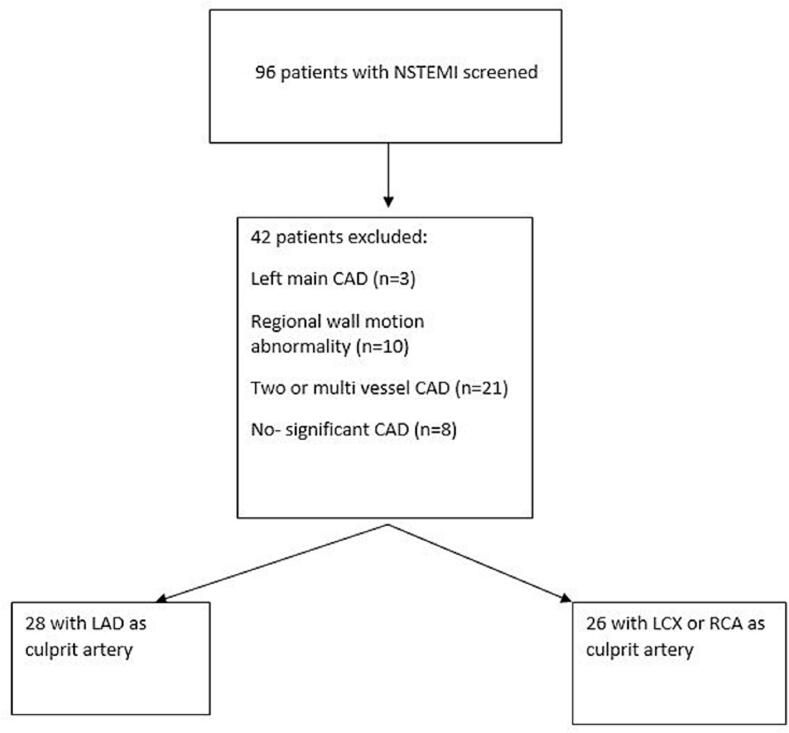
Flowchart showing the patient selection process. A total of 96 patients with NSTEMI were screened. After excluding 3 patients with left main CAD, 10 with regional wall motion abnormalities, 21 with two- or multivessel CAD, and 8 with non- significant CAD, 54 patients were included in the final analysis (NSTEMI: Non-ST-segment elevation myocardial infarction; CAD: Coronary artery disease).

Applying a descriptive-correlational research design, this study was conducted on 96 patients with NSTEMI and LVEF≥50% as well as no RWMA, admitted to the emergency department of our hospital between 2022 and 2023. The patient selection process is summarized in Fig. 1. Among the 96 NSTEMI patients screened, 42 were excluded due to significant left main, two- or multivessel coronary artery disease (CAD), RWMA, or non-significant CAD, leaving 54 patients for the final analysis.

To meet the ethical considerations, the Declaration of Helsinki (DoH) guidelines were observed, and the study received the approval by the Ethics Committee of the mentioned hospital (Ethics code no. IR.MAZUMS.REC.1400.9233).The informed consent form was also obtained from all patients. Of note, NSTEMI was diagnosed according to the presence of angina pectoris and the cardiac troponin I (TnI) levels above the upper normal limits (0.04 ng/ml). The TnI level was thus measured at presentation, and then repeated every 4–6 h for two days [8]. A transthoracic echocardiography was further performed for all patients within the first day of admission, and the data were interpreted by an echocardiographer. Besides, CAG was performed for all patients within 24–48 h of admission. The patients were excluded if they had persistent ST-segment elevation of 0.1 mv or more (0.2 mV in precordial leads V1-V3) in two or more contiguous leads, the TnI levels within the normal limits (<0.04 ng/ml), the anterior ST depression as an indication of posterior STEMI, left main CAD or more than one major epicardial artery with the stenosis≥70% (as the culprit artery could not be determined), previous MI with reference to electrocardiogram (ECG), prior history of coronary artery bypass graft (CABG) or percutaneous coronary intervention (PCI), hemodynamic instability or cardiogenic shock, arrhythmia, heart block, permanent pacemaker or intracardiac defibrillator, history of heart failure, significant valvular heart disease, RWMA, pre-excitation syndrome, poor echocardiographic view, or pregnancy.

All patients underwent CAG during 24–48 h of admission by the Siemens AG (Medical Solutions: Erlangen, Germany). The angiograms were then interpreted by an experienced cardiologist blinded to the patient data. Accordingly, significant CAD was defined if there was the stenosis of more than 50% in the LM artery or over 70% in other coronary arteries [9]. The major epicardial coronary artery, including the LAD, left circumflex (LCX), and right coronary artery (RCA), with the critical stenosis≥70%, was considered as the culprit artery. The patients with two, three, or multivessel CAD and more than one major epicardial artery with significant CAD were excluded from the study because the culprit artery could not be determined. Since only patients with single-vessel CAD were included, the culprit artery was identified based primarily on CAG findings. The artery showing significant CAD with angiographic features consistent with acute plaque rupture, thrombus formation, or impaired distal flow was defined as the culprit vessel. ECG changes and clinical presentation were reviewed to support the angiographic interpretation when applicable.

Hypertension (HTN) was further characterized as systolic blood pressure (SBP) ≥ 140 mmHg and/or diastolic blood pressure (DBP) ≥ 90 mmHg or need for taking antihypertensive drugs [10]. Hyperlipidemia (HLP) was also described as total cholesterol (Chol) levels>200 mg/dl, high-density lipoprotein (HDL)-Chol levels<40 mg/dl in males or < 50 mg/dl in females [11]. Besides, diabetes mellitus (DM) was defined according to the American Diabetes Association (ADA) guidelines, containing fasting blood sugar (FBS) ≥ 126 mg/dl (7.0 mmol/l) or 2-h plasma glucose levels during 75-g oral glucose tolerance test (OGTT) ≥ 200 mg/dl (11.1 mmol/l) or hemoglobin A1c (HbA1c) ≥ 6.5% (48 mmol/mol) or need to take insulin or oral hypoglycemic agents [12]. Moreover, cigarette smoking was identified by face-to-face survey. Body mass index (BMI) was then identified as weight in kg divided by height in m^2^.

### 2.1 Standard 2D-STE

**Fig. 2:**
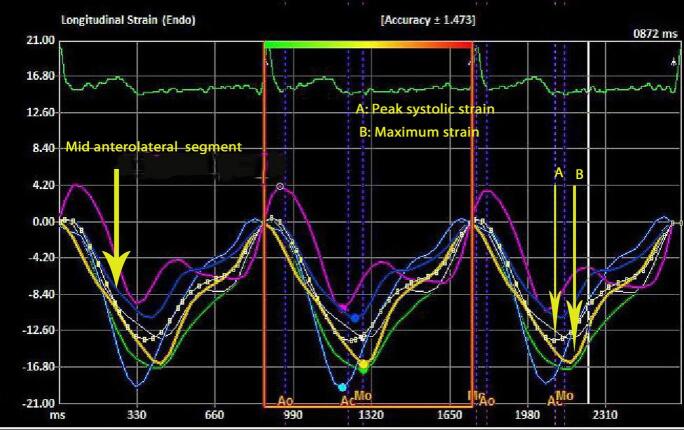
Longitudinal speckle-tracking echocardiography in apical four chamber view displays strain curves in inferoseptal and anterolateral LV myocardial walls by dividing them into three segments consist of basal, mid and apical segments. The PSI is calculated by this formula: (maximum negative strain in cardiac cycle - peak negative systolic strain)/ (maximum negative strain in cardiac cycle) x 100. Yellow vertical arrows display peak negative systolic strain and maximum negative strain in cardiac cycle in mid anterolateral myocardial segment (LV: Left ventricle; PSI: Post systolic index). (For interpretation of the references to colour in this figure legend, the reader is referred to the web version of this article.)

A transthoracic echocardiography was performed on all patients by the ACUSON SC2000 with a 4V1c transducer (Siemens Medical Solutions Inc., Mountain View: CA, the USA) within the first day of admission. All movies and images were accordingly stored on a hard disk for further off-line analysis (using the eSie VVI software) by an echocardiographer blinded to the patient data. The 2D grayscale movies in the apical four-, two-, and three-chamber views (three standard apical views) were then obtained in three cardiac cycles. As well, the peak longitudinal strain values were acquired from the basal, mid, and apical segments of the inferoseptal, anterolateral, inferior, anterior, inferolateral, and anteroseptal walls by tracking the endocardial and epicardial surface areas. The LV peak global longitudinal strain (LVGLS) was further defined as the average value of all 16 myocardial segments [13]. Moreover, PSS was outlined as the myocardial shortening which occurred after aortic valve closure or end-systole (Fig. 2). In addition, PSI was developed from 16 myocardial segments and determined as (maximum strain - peak systolic strain/ maximum strain) x100 (%). If the time to the maximum strain of the segment curve was before end-systole, the segment PSI would be considered zero [14]. The measurements were thus expressed as the average of three consecutive beats. For territorial analysis, left ventricular segments were assigned to the perfusion territory of each major epicardial coronary artery according to a previously published coronary distribution model. [15]. Segments corresponding to the LAD territory included the anterior, anteroseptal, and apical regions, whereas segments assigned to the LCX territory comprised the anterolateral and inferolateral regions. Segments assigned to the RCA territory included the inferoseptal and inferior regions. Regional PSI values were subsequently averaged within each coronary territory for analysis. The PSI values were then averaged from the corresponding segments to reach the mean value for each major epicardial coronary artery territory. Consequently, the averaged PSI values for the LCX and RCA arteries were added together. The left atrial (LA) diameter was also measured from the para-sternal long axis view as the vertical distance between the posterior wall of the aortic root and the posterior LA wall at end-systole [16]. Transmitral pulse-Doppler-derived early and late diastolic velocities (E and A waves) and *E*-wave deceleration time (DT) were additionally determined in the apical four-chamber view by placing the cursor at the mitral valve leaflet tip. The mean tissue-Doppler-derived mitral annulus septal and lateral early diastolic (e’) and peak systolic (s') velocities were accordingly measured by inserting the cursor at the mitral annulus area. Of note, the pulsed-wave Doppler E/A and tissue-Doppler E/e’ ratio were established. Likewise, the M-mode echocardiography in the parasternal long-axis view was utilized to finalize the end-systolic and -diastolic LV internal diameters and end-diastolic interventricular septal and posterior wall thickness by inserting the cursor at the mitral valve leaflet tip. The LV mass index (LVMI) was also computed by the following formula: 0.8 (1.05 [(LVIDd+PWT + IVST)^3^- (LVIDD)^3^]) + 0.6 in which LVIDd, PWT, and SWT indicated the end-diastolic LV internal dimension and posterior and septal wall thickness, respectively [13]. Left ventricular (LV) mass index was calculated in grams per square meter (g/m^2^) and indexed to body surface area. The reproducibility of the STE-derived LVGLS and average PSI were accordingly settled based on repeated measurements in 10 randomly selected patients by echocardiography within 24 h, and the intra-observer correlation coefficients were found to be 0.9 for LVGLS and 0.87 and 0.85 for PSI_LAD_ and PSI_LCX+RCA_, in that order. All echocardiographic acquisitions and analyses, including STE-derived LVGLS and PSI measurements, were performed and interpreted by a single experienced echocardiographer who was blinded to all clinical and angiographic data. This approach ensured methodological consistency and minimized variability; therefore, assessment of inter-observer variability was not applicable to this study design.

### 2.2 Statistical analysis

In this study, the continuous variables were explained as mean ± standard deviation (M ± SD) and the categorical ones were presented as frequency and percentage. Shapiro-Wilk test was also used for the quantitative variables to determine their normality. Besides, independent-samples *t*-test was employed to compare the normally distributed continuous variables and Mann-Whitney *U* test was utilized to compare the continuous ones with non-normal distribution. On the other hand, Chi-square test and Fisher's exact test were applied to compare the categorical variables. Furthermore, logistic regression analysis (LRA) with backward elimination was carried out to find the independent correlation between different variables and culprit artery within the LAD territory during CAG. All relevant clinical and echocardiographic variables were initially entered into a multivariate logistic regression model to identify independent predictors of CAG-confirmed LAD culprit artery. The variables included age, sex, hypertension, diabetes mellitus, hyperlipidemia, LVGLS, E/e' ratio, average PSI_LAD_, and average PSI_LCX+RCA_. A backward stepwise elimination method was then applied to retain variables with statistical significance in the final model. Correspondingly, ROC curve analysis was considered to consider the diagnostic accuracy of average PSI_LAD_ in predicting CAG-identified culprit artery within the LAD territory. The *p*-value less than 0.05 was further judged as the significance level. As a whole, the statistical data were analyzed using the SPSS/PASW Statistics software ver. 18 (SPSS, Chicago, IL, the USA).

## 3 Results

This study included 96 patients with NSTEMI, admitted to the emergency department of our hospital. In total, patients presenting with more than one major epicardial artery affected by significant CAD, RWMA, significant left main involvement, or non-significant CAD were excluded from the study. Among the remaining 54 patients, the culprit artery was the LAD in 28 patients, the LCX in 19, and the RCA in 7 patients. The mean age of the patients was 57.89 ± 12.49 and their mean BMI was equal to 26.82 ± 4.472 kg/m^2^. Out of all patients, 39 (72%) and 15 (28%) cases were men and women, respectively. The most common CAD risk factor was HTN (*N* = 33, 53.2%) followed by DM (*N* = 25, 40.3%), HLP (16, 25.8%), and smoking (*N* = 9, 14.5%), in that order. The patients with an identifiable culprit artery during CAG were then divided into two groups based on the pattern of culprit artery including Group I with culprit artery within the LAD territory and Group II having culprit artery in the RCA or LCX. The demographic characteristics, common cardiovascular risk factors, and angiographic outputs of both groups are presented in Table 1. The study results revealed that the demographic characteristics and common cardiovascular risk factors were not significantly different between both groups. Table 2 illustrates the echocardiographic characteristics of the study groups. The E wave and E/e’ ratio were lower in Group I than Group II (0.58 ± 0.16 m/s vs. 0.68 ± 0.15 m/s, *p* = 0.021 and 7.00 [5.87–9.74] vs. 8.67 [7.27–10.70], *p* = 0.043, respectively). Besides, the average PSI_LAD_ was higher in Group I than Group II (3.00 [0.33–9.92] vs. 0.63 [0.06–5.50], *p* = 0.019).

**Table 1:** Demographic characteristics and cardiovascular risk factors in study groups categorized by culprit artery demonstration by CAG.

		Culprit artery		*P* value
		LAD (*n* = 28)	LCX or RCA (n = 26)	
Age (years)		59.00 ± 9.96	61.46 ± 12.24	0.420
Sex (n, %)	Male	20 (71.4%)	19 (73.1%)	0.893
	Female	8 (28.6%)	7 (26.9%)	
DM (n, %)		15 (53.6%)	13 (46.4%)	0.161
HTN (n, %)		16 (57.1%)	13 (50.0%)	0.599
HLP (n, %)		10 (35.7%)	5 (19.2%)	0.177
BMI (kg/m^2^)		26.49 ± 3.87	26.96 ± 4.24	0.673
Smoking (n, %)		5 (17.9%)	4 (15.4%)	1.000

DM: Diabetes mellitus; HTN: Hypertension, HLP: Hyperlipidemia; BMI: body mass index; CAG: Coronary angiography; LAD: Left anterior descending artery; LCX: Left circumflex artery; RCA: Right coronary artery.

**Table 2:** Echocardiographic characteristics of study groups categorized by culprit artery demonstration by CAG.

	Culprit artery		P value
	LAD (n = 28)	LCX or RCA (n = 26)	
Indexed LVIDd (cm/m^2^)	2.58 ± 0.35	2.64 ± 0.38	0.514
Indexed LVIDs (cm/m^2^)	1.58 ± 284	1.58 ± 0.26	0.957
Indexed LA diameter (cm/m^2^)	2.07 [1.91–2.34]	2.09 [1.86–2.25]	0.579
Indexed LV mass (gr/m^2^)	103.54 ± 38.13210	97.69 ± 38.02	0.579
E wave (m/s)	0.58 ± 0.16	0.68 ± 0.15	0.021
A wave (m/s)	0.68 ± 0.18	0.75 ± 0.16	0.163
DT (ms)	210 [185–265]	204 [182–249]	0.777
e’ (cm/s)	8.10 [6.70–8.10]	7.50 [5.90–9.00]	0.817
s' (cm/s)	8.27 ± 1.06	8.05 ± 1.01	0.450
E/A	0.88 [0.68–1.04]	0.95 [0.65–1.13]	0.327
E/e’	7.00 [5.87–9.74]	8.67 [7.27–10.70]	0.043
LVGLS (%)	−17.05± 3.20	−16.86± 3.39	0 0.836
Average PSI_LAD_	3.00 [0.33–9.92]	0.63 [0.06–5.50]	0.019
Average PSI_LCX+RCA_	2.58 [0.66–7.40]	4.19 [1.45–6.45]	0.272

LVIDd: End diastolic left ventricular internal diameter; LVIDs: End systolic left ventricular internal diameter; LA: Left atrium; LV: Left ventricle, DT: Deceleration time; E/e’ transmitral Doppler early diastolic velocity/mitral annular early diastolic velocity, A wave: transmitral Doppler late diastolic velocity, s mitral annular peak systolic velocity; LVGLS: Left ventricular global longitudinal strain; CAG: Coronary angiography; LAD: Left anterior descending artery; LCX: Left circumflex artery; RCA: Right coronary artery; PSI: Post systolic index.

**Fig. 3:**
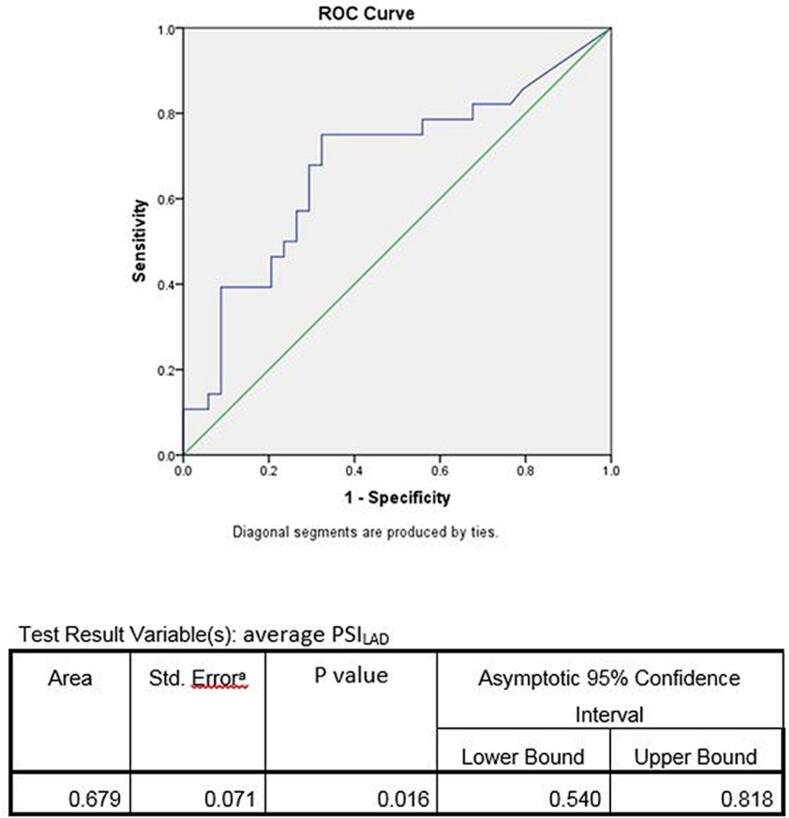
ROC curve for identifying the role of average PSI_LAD_ in predicting angiographic LAD culprit artery (PSI: Post systolic index; LAD: Left anterior descending artery).

The LRA with backward elimination was also conducted on all patients to determine the independent correlation between different demographic and echocardiographic variables and CAG-identified culprit artery within the LAD territory. In the initial logistic regression model, clinical risk factors (age, sex, hypertension, diabetes mellitus, and hyperlipidemia) and echocardiographic variables (LVGLS, E/e', average PSI_LAD_, and average PSI_LCX+RCA_) were included. After backward elimination, only average PSI_LAD_ (odds ratio [OR] 1.115, 95% confidence interval [CI]: 1.005–1.236, *p* = 0.040) could be an independent predictor of the culprit artery within the LAD territory in patients with NSTEMI (Table 3). The ROC curve analysis additionally indicated that the area under the curve (AUC) for the average PSI_LAD_ in predicting CAG-identified culprit artery within the LAD territory was 0.679 (*p* = 0.016; 95% CI [0.540–0.818]) (Fig. 3), so the average PSI_LAD_ ≥ 1.9 had the sensitivity of 68% (95% CI: 55–79%) and specificity of 71% (95% CI: 58–81%) in predicting the CAG-identified LAD culprit artery. To ascertain if the cut-off value was accurate enough to determine the CAG-identified LAD artery involvement, the patients were grouped accordingly. The analysis results showed that more patients in the group with the average PSI_LAD_ ≥ 1.9 had culprit artery within the LAD territory as compared with those with the average PSI_LAD_ < 1.9 (67.9% vs. 29.4%, *p* = 0.003, Table 4).

**Table 3:** The logistic regression analysis determining independent correlation between different variables with CAG-identified culprit lesions in LAD artery.

		B	S.E.	Wald	Sig.	Exp(B)	95% C.I.for EXP(B)	
							Lower	Upper
Step 1^a^	age	−0.010	0.027	0.142	0.706	0.990	0.938	1.044
	sex	−0.771	0.811	0.905	0.342	0.463	0.094	2.266
	HTN	0.260	0.637	0.167	0.683	1.297	0.372	4.520
	HLP	−0.306	0.808	0.143	0.705	0.737	0.151	3.588
	DM	−0.946	0.645	2.154	0.142	0.388	0.110	1.373
	LVGLS	−0.019	0.091	0.046	0.831	0.981	0.820	1.172
	E/e’	−0.125	0.118	1.126	0.289	0.882	0.700	1.112
	Average PSI_LAD_	0.125	0.070	3.206	0.073	1.134	0.988	1.300
	Average PSI_LCX+ RCA_	0.033	0.072	0.209	0.647	1.034	0.897	1.190
	Constant	3.091	3.470	0.793	0.373	22.005		
Step 9^a^	Average PSI_LAD_	0.108	0.053	4.206	0.040	1.115	1.005	1.236
	Constant	−0.654	0.353	3.432	0.064	0.520		

DM: Diabetes mellitus; HTN: Hypertension, HLP: Hyperlipidemia; E/e’ transmitral Doppler early diastolic velocity/mitral annular early diastolic velocity, LVGLS: Left ventricular global longitudinal strain; LAD: Left anterior descending artery; LCX: Left circumflex artery; RCA: Right coronary artery; PSI: Post systolic index.

**Table 4:** Angiographic culprit artery categorized by having or not having average PSI_LAD_ ≥ 1.9.

			Average PSI_LAD_		Total
			<1.9	≥1.9	
LAD	No LAD culprit	Count	24	10	34
		% within LAD	70.6%	29.4%	100.0%
		% within PSI_LAD_ 1.9	72.7%	34.5%	54.8%
		% of Total	38.7%	16.1%	54.8%
	LAD culprit	Count	9	19	28
		% within LAD	32.1%	67.9%	100.0%
		% within PSI_LAD_ 1.9	27.3%	65.5%	45.2%
		% of Total	14.5%	30.6%	45.2%
Total		Count	33	29	62
		% within LAD	53.2%	46.8%	100.0%
		% within PSI_LAD_ 1.9	100.0%	100.0%	100.0%
		% of Total	53.2%	46.8%	100.0%

LAD: Left anterior descending artery; PSI: Post systolic index.

## 4 Discussion

The present study demonstrated that average PSI_LAD_ was significantly higher in patients with LAD culprit lesions than in those with LCX or RCA culprit lesions. Furthermore, average PSI_LAD_ was independently associated with a CAG-identified LAD culprit artery in patients with NSTEMI and preserved LVEF.

Identification of the infarct-related artery (IRA) in NSTEMI remains more challenging than in STEMI because electrocardiographic changes are often nonspecific and regional wall motion abnormalities may be absent or transient [17]. Accurate identification of the culprit vessel is clinically important, particularly in patients with anterior wall infarction, who are at increased risk of adverse in-hospital outcomes, including heart failure and cardiogenic shock [18]. Identifying high-risk patients is also critical for the reason that these patients should be considered for an invasive procedure [19]. Therefore, additional non-invasive tools that assist in culprit vessel localization may have clinical value. Post-systolic shortening (PSS), defined as myocardial shortening occurring after aortic valve closure, can be readily assessed by two-dimensional speckle-tracking echocardiography (2D-STE) [20]. PSS has been recognized as a sensitive marker of myocardial ischemia and ischemic memory, persisting after the resolution of ischemia because of transient myocardial stunning [6], [21]. Previous studies have suggested that PSS may be more sensitive than peak systolic longitudinal strain for detecting regional ischemia [22]. As well, PSI is an index for grading myocardial PSS severity, and defined as maximum strain-peak systolic strain/maximum strain×100 (%). However, PSS is not pathognomonic for myocardial ischemia and may occur in healthy individuals. The normal value for PSI is the median averaged value of 2% (interquartile range [IQR]: 0.7–4.8%) that includes the averaged value of 16 myocardial segments [23]. Therefore, while the association between PSI and myocardial ischemia is well established, the ability of regional PSI to identify the culprit coronary artery in NSTEMI patients with preserved LVEF and without overt wall motion abnormalities has not been well characterized. Our findings suggest that regional PSI_LAD_ may provide complementary information for culprit vessel localization in this diagnostically challenging subgroup.

Increased PSI values in myocardial segments with RWMA accordingly represent viable contracting myocardium [24]. However, the role of PSS has not been well known in patients with NSTEMI with normal LV function. Halvorsrød et al. (2023) evaluated automated tissue Doppler imaging (TDI)-derived myocardial deformation parameters in 84 patients with suspected NSTEMI and investigated their ability to identify acute coronary occlusion. Although TDI-derived strain and post-systolic shortening parameters were not associated with coronary occlusion, speckle-tracking echocardiography-derived strain and PSI demonstrated modest discriminatory ability (AUC 0.66). Importantly, only 17 patients had an acute coronary occlusion, which may have limited the statistical power of the analysis. [25]. In contrast, the present study focused on regional PSI derived from speckle-tracking echocardiography and its association with culprit vessel localization in a highly selected NSTEMI population. Differences in study objectives, patient selection, imaging methodology, and clinical endpoints may explain the variation in findings between the two studies. In this line, Giridharan et al. (2021) investigated 159 patients with suspected unstable angina (UA) without any RWMA. All patients also underwent STE and CAG. LVGLS, pathological PSS presence or absence, PSI_17_ and PSI_12_ (from 6 basal and 6 mid-ventricular segments), and PSI in the LAD, LCX, and RCA territories were further assessed. Furthermore, significant CAD was seen in 54.7% of the patients. The prevalence rate of PSS (62.1% vs. 13.9%), mean PSI_17_ (5.4 vs. 3.3), and PSI_12_ (6.2 vs. 3.7) were significantly higher in those with CAD than the cases without a significant disease. Moreover, multivariate regression analysis (MRA) outcomes showed that PSS and PSI_17_ were predictors of significant CAD. As well, PSS had the sensitivity of 62.1% and the specificity of 86.1% with a positive predictive value of 84.4%. PSI_17_ (AUC: 0.637; *p* = 0.003) and PSI_12_ (AUC: 0.661; *p* = 0.001) also had moderate accuracy in determining significant CAD. The accuracy of regional PSI in identifying the significant lesions in the LAD artery was poor with the AUC of 0.575 (95% CI: 0.484–0.665; *p* = 0.112). Additionally, regional PSI in determining the lesions in the LCX and RCA arteries had only moderate accuracy in finding culprit artery with the AUC of 0.602 (95% CI: 0.493–0.711; *p* = 0.065) and 0.624 (95% CI: 0.522–0.725; *p* = 0.024), respectively. This might be attributable to the larger percentage of patients with multi-vessel disease (56.3%) or the overlap in the segments vascularized based on vessel dominance. Although they also evaluated regional PSI values in the LAD, LCX, and RCA territories, their study population consisted of patients with suspected unstable angina, a lower-risk cohort with a higher prevalence of multivessel disease and without regional wall motion abnormalities. These factors may have reduced the ability of regional PSI to discriminate between culprit coronary territories. In contrast, our study focused on NSTEMI patients with preserved LVEF and single-vessel disease, thereby minimizing potential confounding from overlapping ischemic territories and chronic myocardial remodeling. Furthermore, we assessed regional average PSI_LAD_ by averaging PSI values across LAD-supplied segments, which may reduce segmental variability and improve measurement reproducibility. Under these more controlled conditions, regional average PSI_LAD_ was independently associated with a CAG-identified LAD culprit artery. Taken together, these findings suggest that regional PSI may provide complementary information for culprit vessel localization in selected NSTEMI patients, although further validation in larger and more representative populations is required. As well, Masada et al. (2023) examined 43 patients suspected of intermediate- or low-risk NSTE-ACS, whose echocardiographic images were acceptable for strain analysis. All patients underwent CAG. Among them, 26 cases had CAD and 21 individuals were subjected to PCI. Patients with CAD had higher PSI (25% [20.8–40.3%] vs. 15% [8.0–27.5%], *p* = 0.007). As well, ROC curve analysis showed that PSI > 20% determined the PCI performance (sensitivity = 80.7%, specificity = 70.6%, and AUC = 0.72). Furthermore, the AUC acquired by the Global Registry of Acute Coronary Events (GRACE) risk score was 0.57 (95% CI 0.39–0.75) and then increased to 0.75 (95% CI 0.60–0.90) by adding PSI and LVGLS [26]. In comparison to the present study in which the average PSI value was utilized, they added values from all myocardial segments together to define the total PSI value.

In the other study on 83 patients with UA/NSTEMI and LVEF≥50%, who had undergone CAG and STE, the patients were divided into two groups according to high (≥22) and low (<22) SYNTAX score. The patients with a high SYNTAX score accordingly had a higher cumulative PSI and LVGLS than those with a low score. Among different variables, only PSI was an independent predictor of the high SYNTAX score. They further suggested that cumulative PSI might predict more complex and extensive CAD among patients with UA/NSTEMI [19].

In contrast to previous studies that mainly assessed global or segmental PSS or PSI values, the present study introduced a regional average PSI_LAD_, calculated by averaging the PSI values of the left ventricular segments supplied by the LAD artery. This region-based approach provides a more territory-specific assessment of ischemia, reflecting the functional impact of culprit artery within the corresponding coronary distribution rather than isolated segmental changes. By directly correlating the regional average PSI_LAD_ with CAG findings, our study demonstrates that PSI, when analyzed regionally, can serve as a sensitive and non-invasive indicator of LAD involvement in patients with NSTEMI and preserved LVEF. Clinically, the regional average PSI_LAD_ offers additional diagnostic value by enabling early recognition of LAD-related ischemia even in the absence of overt wall-motion abnormalities, thereby supporting more accurate risk stratification and timely decision-making regarding coronary angiography or revascularization.

Another methodological consideration in our study relates to the influence of coronary dominance on regional myocardial deformation analysis. Because the perfusion territories of the RCA and LCX can vary considerably among individuals depending on dominance pattern, strict assignment of segments to a single coronary artery may not fully reflect the true physiological distribution. To mitigate this limitation, we combined the PSI values of the RCA and LCX territories when evaluating non-LAD regions. This approach provided a more balanced representation of the inferior and inferolateral walls, which are often variably supplied by either artery, and thereby reduced potential misclassification bias arising from dominance-related anatomical differences. Nevertheless, we acknowledge that a patient-specific anatomical mapping based on coronary angiography would further refine territorial assignment and should be explored in future studies.

Regional PSI may provide additional information for identifying LAD-related ischemia in a clinical setting where conventional echocardiographic abnormalities are absent**.** However, due to its moderate diagnostic performance, it should not be considered a standalone diagnostic tool. Rather, it may serve as a complementary echocardiographic parameter alongside established clinical, electrocardiographic, biomarker, and imaging assessments. Whether PSI provides incremental diagnostic value beyond current risk stratification approaches requires further investigation.Given the known prognostic importance of LAD-related infarction, early identification of LAD involvement may facilitate risk stratification and clinical decision-making.

Because patients with multivessel disease, reduced LVEF, and overt RWMA were excluded, and because the study included a small number of patients, the findings should be considered hypothesis-generating and applicable primarily to a selected NSTEMI population.Further studies including broader and more representative NSTEMI cohorts are needed to determine the clinical utility and generalizability of regional PSI.

### 4.1 Limitations

Several limitations should be acknowledged. First, this was a single-center study with a relatively small sample size, which may have limited statistical power and reduced the generalizability of the findings. Therefore, the observed associations should be considered exploratory and require confirmation in larger multicenter cohorts.

Second, the study population was highly selected, including only patients with preserved LVEF and excluding those with multivessel disease, prior myocardial infarction, and overt regional wall motion abnormalities. Although these criteria minimized potential confounding and enabled a more focused assessment of the relationship between regional PSI and culprit vessel location, they limit the applicability of the findings to the broader NSTEMI population encountered in routine clinical practice.

Third, coronary territories were assigned according to a theoretical segmental model that does not account for individual variations in coronary anatomy or coronary dominance. Such variability may have affected the accuracy of territorial assignment and contributed to the moderate diagnostic performance observed. Future studies integrating patient-specific coronary angiographic mapping with segmental PSI analysis may improve diagnostic precision.

Fourth, electrocardiographic findings were not systematically analyzed. Consequently, the incremental diagnostic value of regional PSI beyond conventional ECG-based approaches for culprit vessel localization could not be determined. Future investigations should evaluate the combined utility of electrocardiographic and speckle-tracking echocardiographic parameters in patients with NSTEMI.

Finally, the cross-sectional design precluded assessment of clinical outcomes and prognostic implications. In addition, post-systolic shortening is not specific to acute ischemia and may also occur in non-ischemic myocardial conditions and even in healthy individuals. Variations in the timing of echocardiographic assessment relative to the ischemic event, differences in ischemic burden, and the potential influence of loading conditions may have affected PSI measurements. Accordingly, larger prospective studies with longitudinal follow-up are warranted to validate these findings and clarify their clinical and prognostic significance.

In our study, ROC curve analysis demonstrated that regional average PSILAD had an AUC of 0.679 (95% CI: 0.540–0.818, *p* = 0.016), indicating a statistically significant but only moderate ability to identify an LAD culprit lesion in patients with NSTEMI and preserved LVEF. This level of discrimination is consistent with previous reports evaluating strain-based parameters for ischemia detection. Several factors may explain the modest diagnostic performance observed. First, overlap between the perfusion territories of the major coronary arteries, particularly within border zones between the LAD, LCX, and RCA, may attenuate regional strain differences and reduce the discriminatory value of PSI for a single vascular territory. Second, myocardial deformation parameters are influenced by physiological factors beyond ischemia, including preload and afterload conditions, which may vary among patients despite preserved LVEF. In addition, microvascular dysfunction or unrecognized prior myocardial injury may contribute to post-systolic shortening independent of the acute culprit artery. Together, these factors likely contributed to the moderate diagnostic accuracy of regional PSILAD in the present study.

## 5 Conclusion

To our knowledge, this study is among the first to demonstrate that regional average PSI_LAD_ is independently associated with a CAG-identified LAD culprit artery in NSTEMI patients with preserved LVEF and no overt regional wall motion abnormalities. These findings suggest that regional PSI_LAD_ may serve as a complementary echocardiographic marker for culprit vessel localization in selected patients. However, given the modest diagnostic performance and the highly selected study population, further prospective studies in larger and more representative NSTEMI cohorts are required before routine clinical application can be recommended.

## CRediT authorship contribution statement

**Aliasghar Farsavian:** Writing – review & editing, Visualization, Validation, Investigation, Data curation, Conceptualization. **Maryam Nabati:** Writing – review & editing, Writing – original draft, Visualization, Validation, Supervision, Methodology, Investigation, Formal analysis, Data curation, Conceptualization. **Mehrdokht Sadrolodabai:** Writing – review & editing, Visualization, Methodology, Investigation, Data curation. **Saeed Kavousi:** Writing – review & editing, Visualization, Investigation, Data curation, Conceptualization.

## Funding

There was no external funding source.

## Declaration of competing interest

The authors declare there is no conflict of interest.

## Data Availability

The datasets used and/or analyzed during this study are available from the corresponding author on reasonable request.
